# Neovascular Prostate-Specific Membrane Antigen Expression Is Associated with Improved Overall Survival under Palliative Chemotherapy in Patients with Pancreatic Ductal Adenocarcinoma 

**DOI:** 10.1155/2017/2847303

**Published:** 2017-10-25

**Authors:** Katharina Stock, Konrad Steinestel, Rebekka Wiesch, Jan-Henrik Mikesch, Anna Hansmeier, Marcel Trautmann, Nora Beller, Jan Rehkämper, Eva Wardelmann, Birthe Heitkötter, Wolfgang Hartmann, Jan Sperveslage, Sebastian Huss

**Affiliations:** ^1^Gerhard-Domagk-Institute of Pathology, University Hospital Münster, Münster, Germany; ^2^Institute of Pathology and Molecular Pathology, Bundeswehrkrankenhaus Ulm, Ulm, Germany; ^3^Department of Medicine A, University Hospital Münster, Münster, Germany

## Abstract

**Aims:**

Expression of PSMA (prostate-specific membrane antigen) has been demonstrated in various cancers, including pancreatic ductal adenocarcinoma (PDAC). However, PSMA expression in PDAC-associated neovasculature has so far not been systematically analyzed.

**Methods and Results:**

We analyzed PSMA expression in 81 PDAC tissue samples from 61 patients. Microvessel density (MVD) was assessed by software-based image analysis and showed a mean MVD of 63.7 microvessels/0.785 mm^2^. PSMA was practically absent in tumor tissue (5.3%) and PDAC cell lines (0/7) but could be detected in tumor-associated neovasculature in 53.2% of cases. There was no association between neovascular PSMA expression and clinicopathological tumor characteristics. Samples with PSMA+ neovasculature showed increased MVD; however, this result was not statistically significant (*p* > 0.05). Presence of PSMA+ neovessels correlated with overall survival under palliative chemotherapy (894 versus 400 days; HR 0.42; 95% CI: 0.12 to 0.87; *p* < 0.05).

**Conclusion:**

PSMA expression in tumor-associated neovasculature is a common feature and associated with improved overall survival under palliative chemotherapy in PDAC. Our results point towards a possible association between PSMA expression and response to therapy which might be based on enhanced intratumoral bioavailability of systemic chemotherapy.

## 1. Introduction

Worldwide, pancreatic ductal adenocarcinoma (PDAC) is the fourth leading cause of death from cancer, and both incidence and mortality rates have been on the rise in the last years [1]. Although the molecular mechanisms behind PDAC tumorigenesis and progression are increasingly understood, there have only been modest improvements in 5-year survival rates, conveying to it the doubtful honor of being one of the most aggressive human malignancies [2]. In a curative approach, primary surgery followed by adjuvant chemotherapy remains standard of care [3]. The majority of patients with pancreatic cancer, however, are diagnosed in a palliative situation (metastatic and/or inoperable tumor) or eventually relapses after initial curative approach. These patients receive 5-FU or Gemcitabine-based chemotherapies. However, poor or short-lived responses of the majority of the patients remain the major problem treating advanced pancreatic cancer, and it has been suggested that poor response to Gemcitabine therapy may be due to ineffective delivery of chemotherapy to cancer cells [4].

Prostate-specific membrane antigen (PSMA), a 100 kDa type II-transmembrane glycoprotein with folate hydrolase and neurocarboxypeptidase activity, is expressed in prostate epithelium and upregulated on the surface of prostatic adenocarcinoma cells [5, 6]. This is of diagnostic and therapeutic relevance, since PSMA-based imaging technologies for the detection of metastatic disease as well as PSMA-based radioligand therapy regimens have been established and are in clinical use [6–9].

In recent years, it has been shown that PSMA is also expressed in the endothelium of tumor-associated neovasculature of breast, lung, thyroid, and urothelial cancer, where its enzymatic activity may be involved in malignancy-driven neoangiogenesis [10–15]. PSMA might in fact act as part of an autoregulatory loop involving *β*1-integrin and p21-activated kinase 1 (PAK1) and facilitate endothelial cell invasion through the extracellular matrix (ECM) by interacting with the cytoskeleton via integrin signaling and actin-binding protein Filamin A [16, 17]. This phenomenon is not limited to epithelial tumors, as our own group has previously shown PSMA upregulation in tumor-associated neovasculature of several high grade sarcomas such as synovial sarcoma [18]. In PDAC, PSMA expression on pancreatic cancer cells has been observed [19]; however, to the best of our knowledge, PSMA in tumor-associated neovasculature of pancreatic neoplasms has so far not been investigated.

In the present study, our aim was to assess PSMA expression levels in PDAC as well as in tumor-associated neovasculature of tissue samples from patients who had received palliative chemotherapy with Gemcitabine. We tested for possible correlations between neovascular PSMA expression levels and biological tumor characteristics, microvessel density, response to Gemcitabine, and overall survival. Our results might ultimately help to predict the response to Gemcitabine in patients with aggressive PDAC.

## 2. Materials and Methods

### 2.1. Patients and Clinicopathologic Data

In total, 81 formalin-fixed, paraffin-embedded PDAC tissue samples from 61 patients were included in the study. Detailed clinicopathological data were retrieved from the respective pathology reports/clinical records and are summarized in Table 1. The use of the human tissue samples was approved by the ethics committee of the University of Münster (Approval Number 2015-102-f-S).

### 2.2. KRAS Mutation Analysis by Sanger Sequencing/Next-Generation Sequencing

Amplification of* KRAS* Exon 2 PCR products was carried out with the following PCR primers: 5′-GTCACATTTTCATTATTTTTATTATAAGGCCTG-3′ and 5′-CCTCTATTGTTGGATCATATTCGTCCAC-3′. PCRs were conducted with the Gene Amp™ Fast PCR Master Mix (Life Technologies, Carlsbad, CA). Sequencing PCRs were performed with an initial denaturation at 96°C for 1 min for 25 cycles at 96°C for 10 s, 50°C for 5 s, and 60°C for 1.15 min using the BigDye® Terminator v3.1 Mix Cycle Sequencing Kit (Life Technologies) and the gene specific primers mentioned above. After purification with MultiScreen®-HV Plates (Merck Millipore) and Sephardi G-50 (GE Healthcare, Chalfont St Giles, UK) the PCR products were sequenced using a 96-capillary 3730xl DNA Analyzer (Life Technologies).

In addition, samples with low tumor cell content were analyzed by more sensitive next-generation sequencing under the use of a Custom GeneRead DNASeq Panel (Qiagen, Hilden, Germany) consisting of 189 amplicons for mutation analysis of 19 cancer-related genes following the manufacturer's instructions. In short, genomic DNA was quantified and amplified with 4 primer pools. After pooling and purification of PCR products from the same patients, enzymatic modifications (end repair, A-addition, and adapter ligation) were performed with the GeneRead DNA Library I Core Kit (Qiagen) following the manufacturer's instructions. After additional purification with Agencourt AMPure XP beads (Beckman Coulter), a Library PCR amplification with the GeneRead DNA Amp Kit (Qiagen) was conducted. After a final purification with Agencourt AMPure XP beads (Beckman Coulter), Library PCR products were quantified, diluted, pooled, and sequenced using the MiSeq™ V2 reagent kit on a MiSeq instrument (Illumina, San Diego, CA, USA). Data were exported as FASTQ files and analyzed with the CLC Biomedical Genomic Workbench (Qiagen).

### 2.3. Construction of PDAC TMA and Immunohistochemistry

Four different tissue microarrays (TMAs) containing at least two representative cores (core diameter 1 mm) per case were designed after selection of tumor areas by two pathologists (KS and JR) before and after TMA construction. Duplicates were taken from the same paraffin block and if possible, from the same tumor area (tumor/metastasis centre), adding to a total of 162 cores from 81 blocks and 61 patients. Presence of tumor cell budding had previously been assessed on full slides using the method described by Karamitopoulou et al. [20]. 4 *μ*m thick slides were cut and stained using a mouse monoclonal anti-PSMA antibody (clone 3E6, Ventana, Germany, 1 : 50 dilution) and monoclonal anti-CD34 antibody (clone QBEnd10, Ventana, Germany, ready to use concentration of 0.8 *μ*g/ml) as previously described [18]. In brief, sections were deparaffinized in xylene and rehydrated through graded ethanol at room temperature. Incubation with the primary antibodies was performed for 30 minutes at room temperature. After washing, the sections were incubated with biotinylated secondary antibodies. Immunoreactions were visualized using a 3-amino-9-ethylcarbazole as a substrate (Ventana Optiview DAB IHC detection Kit, Ref: 760-700, Germany). Prostate carcinoma tissue sections served as a positive control for PSMA immunostaining.

### 2.4. Assessment of PSMA Expression

**Figure 1:**
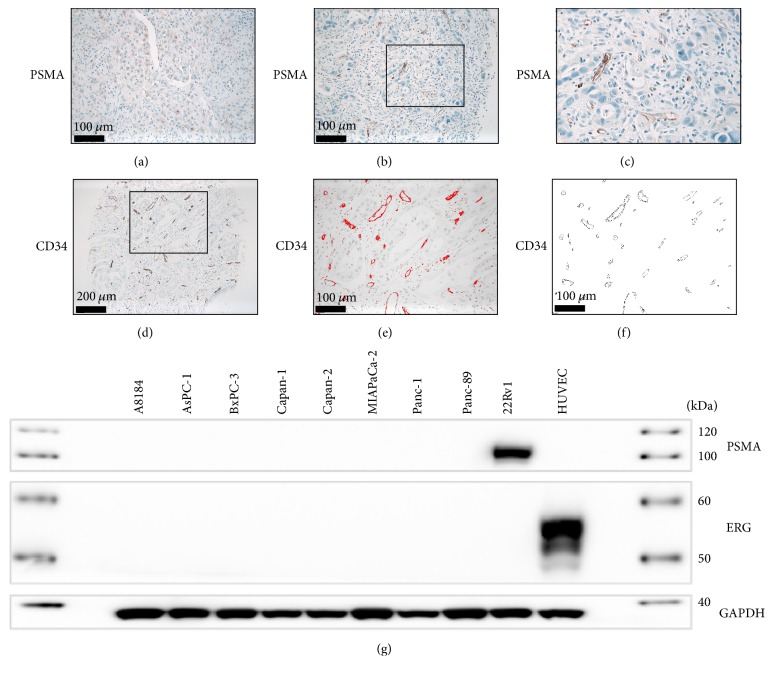
(a) Weak immunostaining for PSMA in PDAC tumor cells. ((b) and (c)) Strong PSMA expression in tumor-associated neovasculature. ((d)–(f)) Software-based assessment of microvessel density (MVD) in CD34-stained tissue microarrays. (g) Strong expression of PSMA in 22Rv1 prostate adenocarcinoma cells, but not in PDAC or HUVEC endothelial cell lines.

PSMA expression was evaluated independently by two pathologists (BH and KS) on immunostained TMA sections. Pathologists were blinded with respect to clinical data. Tumor cells and associated neovascular endothelium were analyzed separately, and the identity of vascular structures was confirmed by CD34 expression, a common marker for endothelial cells [21–23]. IHC for CD31 was evaluated as an internal control and showed no significant differences in staining intensity or MVD (see below). Any PSMA reactivity in either tumor cells or neoplastic vessels was considered positive (Figures 1(a) and 1(b)). Staining intensity was scored semiquantitatively as negative (0), weak (1 = barely perceptible staining at high power (400x) magnification), moderate (2 = readily apparent at medium power (100x) magnification), or strong (3 = readily apparent at low power (40x) magnification). For tumor-associated neovasculature, the PSMA+/CD34+ vessel ratio (<0.05, 0.05–0.1, or >0.1) was assessed as previously described [18].

### 2.5. Slide Scanning and Automated Image Analysis

For automated image analysis and quantification of microvessel density (MVD), CD34-stained TMAs were scanned using a digital slide scanner (Leica SCN400, Leica Microsystems, Wetzlar, Germany). Quantification of MVD in 1 mm TMA cores was performed using ImageJ (NIH, Maryland, USA) using a slight modification of a protocol that has previously been described [24]. In short, (1) channels were split, (2) outlinings of CD34-positive vessels were detected, (3) objects meeting the predefined criteria (size, circularity) were counted automatically for the whole TMA core (0.785 mm^2^) (Figures 1(e) and 1(f)).

### 2.6. Western Blotting of PDAC Cell Lines

PDAC cell lines (BxPC-3, Capan-1, Capan-2, MIA PaCa-2, and Panc-1) and HUVECs were obtained from DSMZ (Braunschweig, Germany). The PDAC cell lines AsPC-1 and Panc89 and the Prostatic adenocarcinoma cell line 22Rv1 were, respectively, gifts from Dr. Bence Sipos (Tübingen) and Dr. Christof Bernemann (Münster). All pancreatic adenocarcinoma cell lines were grown in RPMI 1640 or DMEM supplemented with 10% FCS (FBS; Life Technologies, Carlsbad, CA, USA) under standard cell culture conditions (37°C, 5% CO_2_) to 70% confluence. HUVECs were cultivated in Endothelial Cell Growth Medium w/SupplementMix (fetal calf serum 0.02 ml/ml, endothelial cell growth supplement 0.004 ml/ml, 0.1 ng/ml recombinant human epidermal growth factor, 5 ng/ml recombinant human basic fibroblast growth factor, heparin, and hydrocortisone) under standard cell culture conditions (37°C, 5% CO_2_) to 70% confluence. Protein was extracted using cell lysis buffer (Cell Signaling Technologies, Danvers, MA, USA) following the manufacturer's instructions. Western blotting was performed according to standard methods using a rabbit monoclonal anti-PSMA antibody (clone D4S1F, Cell Signaling Technology, Danvers, MA, USA, dilution 1 : 1000). Anti-ERG (clone EP111, Dako/Agilent, Santa Clara, CA, USA, dilution 1 : 500) was used as a positive control for HUVECs. GAPDH was used as loading control (clone D16H11, Cell Signaling Technology, Danvers, MA, USA, dilution 1 : 1000).

### 2.7. Statistical Analysis

Data are expressed as mean +/− standard derivation. Correlations between categorical variables were assessed using Chi-Square/Fisher's Test, while differences between continuous variables were assessed using *t*-test/ANOVA. Survival analyses were performed using Log-Rank/Gehan-Breslow-Wilcoxon test, respectively. GraphPad Prism (GraphPad, La Jolla, USA) was used for all statistical calculations, and statistical significance was achieved with a *p* value of <0.05.

## 3. Results

### 3.1. Clinicopathological Data

In total, 162 tissue cores from 81 tumor specimens (primary tumors and/or metastases) were included on the TMA. These were derived from primary tumors and metastases from a total of 61 patients; detailed clinicopathological data is given in Table 1. Of note, none of the tumors was classified as well-differentiated (G1). 86.9% of patients had been given palliative chemotherapy, while 13.3% had received radiotherapy in a palliative setting.

### 3.2. PSMA Expression in Tissue Samples and Cell Lines

Weak to moderate PSMA immunoreactivity of PDAC tumor cells was detected in 5.3% of cases (Figure 1(a)), while PSMA expression in tumor-associated neovasculature (cutoff PSMA+/CD34+ vessel ratio > 0.05) could be detected in 63.2% (weak/moderate in 33.4% and strong in 29.8% of cases, resp.) (Figures 1(b) and 1(c) and Table 1). In cases with PSMA-positive neovessels, the PSMA+/CD34+ vessel ratio was 0.05–0.1 in 31.6% and >0.1 in 31.6%, respectively. In cases with strong neovascular PSMA staining intensity, the PSMA+/CD34+ vessel ratio was also significantly higher compared to cases with weak or moderate staining intensity (*p* = 0.0036, *t*-test). PSMA could be detected in lysate from 22Rv1 prostate adenocarcinoma cells, but not in the 7 tested PDAC cell lines (Figure 1(g)).

### 3.3. MVD

Software-based analysis of microvessel density (MVD) revealed a mean (median) MVD of 63.7 (60.75) per 1 mm core (0.785 mm^2^) (Figures 1(d)–1(f) and Table 1).

### 3.4. Correlations between PSMA Expression, Biological Parameters, and Clinicopathological Data

**Figure 2:**
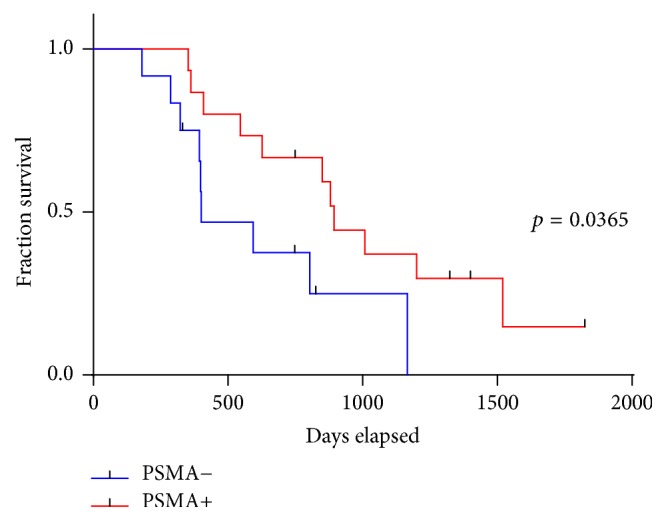
Overall survival is significantly improved in patients with PSMA-positive microvessels.

There was no significant association between neovascular PSMA expression and clinical or biological parameters such as TNM/UICC/AJCC stage,* KRAS* mutation status, primary or metastatic tumor, or tumor cell budding in the investigated cohort (all *p* > 0.05,* Chi-Square/Fisher's exact test*). However, the presence of PSMA-expressing neovessels correlated with overall survival (OS: 894 versus 400 days; HR 0.42; 95% CI: 0.12 to 0.87; *p* = 0.0365;* Log-Rank/Gehan-Breslow-Wilcoxon test*), with significantly shorter OS for patients without PSMA+ neovessels (Figure 2). While event-free survival (EFS) was slightly longer for PSMA-positive cases (190 versus 181 days), this result did not reach statistical significance (HR 0.95; 95% CI: 0.34 to 2.62; *p* > 0.05). Neither the neovascular PSMA staining intensity nor the percentage of PSMA+ vessels correlated with MVD in the investigated tissue cores (all *p* > 0.05, *t*-test and* linear regression analysis*, resp.).

## 4. Discussion

In the palliative treatment setting of pancreatic ductal adenocarcinoma (PDAC), 5-FU or Gemcitabine-based chemotherapies are administered as first-line regimens. However, the majority of the patients show poor and/or short-lived responses to systemic chemotherapies only, a problem that might at least in part be due to an ineffective drug delivery to pancreatic cancer cells [4, 25]. PSMA expression in tumor-associated neovasculature has been described in a variety of solid tumors and is suggested to be involved in (neo)angiogenetic processes [10–15]. Therefore, the aim of this study was to analyze PSMA expression patterns in PDAC and to evaluate a possible correlation between PSMA expression, microvascular density, and response to Gemcitabine treatment in a cohort of 81 patients with histologically confirmed PDAC under palliative chemotherapy.

We could confirm weak PSMA expression in PDAC tumor cells only in a small minority of cases (5.3%) and in none of the tested PDAC cell lines. This is in contrast to a previous study indicating PSMA as a marker of pancreatic tumor cells [19]; however, another large-scale PSMA expression analysis including a multitude of benign and malignant tissue samples and a designated focus on antibody specificity also failed to detect moderate or strong PSMA levels in PDAC [26]. In that study, the authors found weak PSMA immunoreactivity in 2% of cases (1/48), comparable to the results we report here. In the Human Protein Atlas (http://www.proteinatlas.org/), weak PSMA immunostaining is reported in 8% of cases (1/12) for both of the evaluated antibodies (clones 107-1A4 and 1D6) [27]. Taken together, while certain differences in immunostaining might be attributable to different clones or IHC protocols that were used in these studies, we would not recommend PSMA as a useful IHC marker for PDAC tumor cells.

With respect to the tumor-associated neovasculature, we detected PSMA in almost two-thirds of the cases, with weak/moderate immunoreactivity in 33.4% and strong immunoreactivity in 29.8% of cases, respectively. This is in line with findings from breast, lung, thyroid, and urothelial cancer; moreover, our own group reported strong PSMA expression in tumor-associated neovasculature of a significant percentage of rhabdomyosarcomas, malignant peripheral nerve sheath tumors, synovial sarcomas, and undifferentiated pleomorphic sarcomas [18].

The exact role of PSMA in tumor-associated (neo)angiogenesis is so far unclear. While it has been shown that angiogenesis is severely impaired in PSMA-null animals, the relevance of PSMA seems not to be restricted to proteolytic cleavage of the extracellular matrix (ECM) [16]. In fact, PSMA is crucial for the activation of the laminin-binding integrin *β*1 as well as of PAK-1, while being linked to the cytoskeleton via the actin-binding protein filamin A [16]. This interaction establishes an autoregulatory loop between ECM-triggered PSMA activation, integrin signaling, and PAK activation and may ultimately regulate endothelial adhesion and invasion. This is of special interest since prominent stromal fibrosis is a common feature in PDAC and has been associated with the regulation of angiogenesis, PDAC tumor cell survival, and metastasis [28]. A prominent angiogenic response in hypoxic PDAC tissue has been demonstrated convincingly [29, 30]. Therefore, it is well conceivable that hypoxia and/or tumor-derived angiogenetic factors enhance PSMA-mediated angiogenesis in PDAC. In that model, PSMA would facilitate ECM cleavage and/or endothelial cell adhesion, thus contributing to vascular outgrowth and (neo)vascular tube formation.

We could show that PSMA expression in tumor-associated neovasculature correlates significantly with OS in PDAC patients under palliative chemotherapy. A limited number of patients and, as a possible consequence of that, borderline statistical significance has to be stated as a clear limitation of the present manuscript. However, it has also been previously proposed by other authors that poor response to Gemcitabine in PDAC patients might be due to ineffective delivery of systemically administered chemotherapy to cancer cells [4]. The prognostic relevance of MVD is still under debate, because both adverse and favorable prognostic relevance of intratumoral vascular density has been described [31, 32]. These seemingly contradictory results may be due to the fact that MVD is of special relevance only in patients under chemotherapy, since enhanced tumor perfusion might improve the local availability of systemically administered chemotherapy. Such association has previously been shown in breast cancer and, interestingly, chemotherapy seems to reduce the MVD in breast tumors [33, 34]. This effect might also account for the fact that we were unable to show a statistically significant association between PSMA expression and MVD in our sample set.

Finally, since PSMA-targeted radioligand therapy is in routine use for prostate cancer, intratumoral PSMA-positive neovessels might be a promising target for antiangiogenic therapies [6–9]. Further experimental studies are warranted to evaluate a possible use for PSMA-coupled radioligands in this setting.

Taken together, our study shows that PSMA is expressed in tumor-associated neovasculature in the majority of pancreatic adenocarcinomas, but only a small percentage of pancreatic tumor cells. PSMA might be associated with increased neoangiogenesis, and PSMA expression is associated with overall survival of PDAC patients under palliative chemotherapy. PSMA expression might thus be a marker for enhanced tumor perfusion and intratumoral bioavailability of systemically administered chemotherapy regimens. Moreover, PSMA-expressing neovessels might represent promising targets for antiangiogenic therapy using PSMA-coupled radioligands.

## Figures and Tables

**Table 1:** Clinicopathological data of the study cohort.

	*N* (% of evaluated samples)	% of all patients/samples
Number of patients	61	
Number of samples	81	
Age (years)		
Median	65	
range	39–84	
Sex		
Male	40 (65.6)	
Female	21 (34.4)	
Histologic grade (*n* = 67)		
Well-differentiated/G1	0	
Moderately differentiated/G2	38 (56.7)	46.9
Poorly differentiated/G3	29 (43.3)	35.8
UICC stage (*n* = 43)		
IA	0	
IB	1 (2.3)	1.6
IIA	8 (18.6)	13.1
IIB	13 (30.2)	21.3
III	7 (16.3)	11.5
IV	14 (32.6)	22.9
Tissue of origin (*n* = 81)		
Primary tumor	35 (43.2)	43.2
Metastasis	46 (56.8)	56.8
Smoking habits (*n* = 40)		
Smoker	8 (20)	13.1
Nonsmoker/never smoked	32 (80)	52.5
Diabetes (*n* = 42)		
Diabetic	17 (40.5)	27.9
Nondiabetic	25 (59.5)	41
Adjuvant CTx (Gemcitabine; *n* = 44)		
Yes	21 (47.7)	34.4
No	23 (52.3)	37.7
Palliative CTx (*n* = 61)		
Yes	53 (86.9)	86.9
No	8 (13.1)	13.1
Palliative RTx (*n* = 45)		
Yes	6 (13.3)	9.8
No	39 (86.7)	63.9
*KRAS* codon 12/13 mutation (*n* = 56)		
Yes	32 (57.1)	52.5
No	24 (42.9)	39.3
PSMA expression in tumor cells (*n* = 57)		
Absent	54 (94.7)	88.5
Weak/moderate	3 (5.3)	4.9
Strong	0	
PSMA expression in tumor-associated neovasculature (*n* = 57)		
Absent	21 (36.8)	34.4
Weak/moderate	19 (33.3)	31.1
Strong	17 (29.8)	27.9
% of PSMA-positive blood vessels (*n* = 57)		
<5%	21 (36.8)	34.4
5–10%	18 (31.6)	29.5
>10%	18 (31.6)	29.5
